# Supplementary material for: Cyclodextrin–Amphiphilic Copolymer Supramolecular Assemblies for the Ocular Delivery of Natamycin
Source: Nanomaterials (Basel). 2019 May 15;9(5):745. doi: 10.3390/nano9050745 (PMC6566826; doi:10.3390/nano9050745)
Supplement: Supplementary file 1 [file nanomaterials-09-00745-s001.pdf]

Supplementary Information

Cyclodextrin–Amphiphilic Copolymer Supramolecular Assemblies for the Ocular Delivery of Natamycin

Blanca Lorenzo-Veiga [1], Hakon Hrafn Sigurdsson [1], Thorsteinn Loftsson [1] and Carmen Alvarez-Lorenzo [2,*]

[1] Faculty of Pharmaceutical Sciences, University of Iceland, Hofsvallagata 53, IS-107 Reykjavik, Iceland; blv3@hi.is (B.L.-V.); hhs@hi.is (H.H.S.); thorstlo@hi.is (T.L.)

[2] Departamento de Farmacología, Farmacia y Tecnología Farmacéutica, R+D Pharma Group (GI-1645), Facultad de Farmacia and Health Research Institute of Santiago de Compostela (IDIS), Universidade de Santiago de Compostela, 15782 Santiago de Compostela, Spain

* Correspondence: carmen.alvarez.lorenzo@usc.es; Tel.: +34-881-815-239

Table S1. Properties of non-loaded Soluplus and Pluronic P103 micelle dispersions in 0.9% NaCl at 25 °C (n.d.= non detectable).

Polymer concentration (%*w*/*v*)	Soluplus				Pluronic P103			
	pH	Mean diameter (nm)	PDI	Mean Zeta potential (mV)	pH	Mean diameter (nm)	PDI	Mean Zeta potential (mV)
5	3.45	73.66 ± 0.19	0.084 ± 0.004	–0.02 ± 0.05	6.30	14.77 ± 0.11	0.140 ± 0.023	–0.02 ± 0.05
4	3.54	71.04 ± 1.10	0.079 ± 0.006	–0.16 ± 0.08	6.33	15.47 ± 0.18	0.125 ± 0.019	–0.16 ± 0.08
3	3.60	69.41 ± 0.28	0.075 ± 0,005	–0.11 ± 0.07	6.26	15.44 ± 0.03	0.069 ± 0.016	–0.11 ± 0.07
2	3.69	67.55 ± 0.57	0.055 ± 0,012	–0.16 ± 0.19	6.25	17.21 ± 0.70	0.185 ± 0.016	–0.16 ± 0.19
1	3.89	69.27 ± 1.45	0.106 ± 0,034	–0.18 ± 0.08	6.24	23.81 ± 1.52	0.224 ± 0.027	–0.18 ± 0.08
0.1	4.86	68.04 ± 1.57	0.184 ± 0.066	–0.25 ± 0.04	5.70	n.d.	n.d.	–0.25 ± 0.04
0.01	5.20	70.00 ± 0.99	0.130 ± 0.034	–0.03 ± 0.18	5.57	n.d.	n.d.	–0.03 ± 0.18

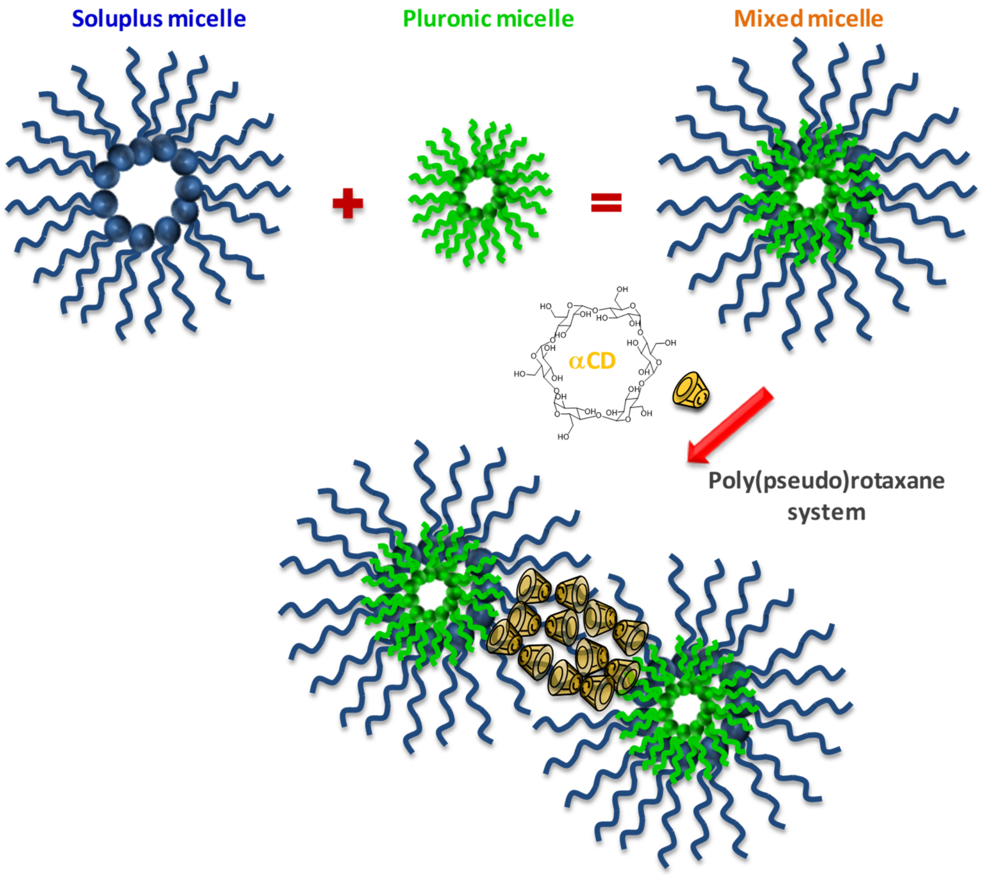

Figure S1. Structure of single and mixed nanomicelles formed by self-assembly of the amphiphilic block copolymers, and of the CD-based poly(pseudo)rotaxanes.

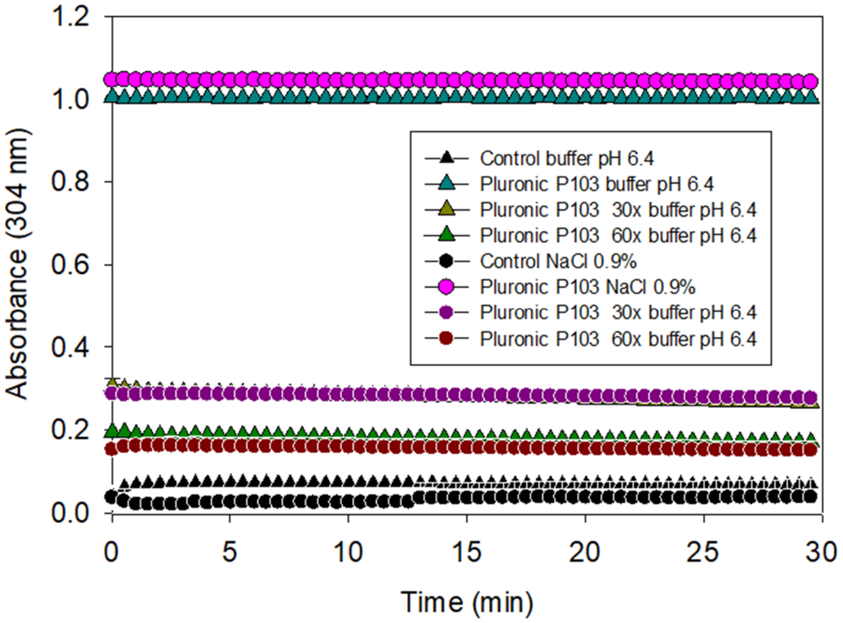

Figure S2. Evolution of the absorbance of natamycin-loaded Pluronic P103 (10%) nanomicelle formulations before (the signal was saturated) and after 30-fold and 60-fold dilution, respectively, in 0.9% NaCl or pH 6.4 buffer. Controls refer to unloaded Pluronic P103 (10%) nanomicelles. Error bars refer to the standard deviations (n = 3).

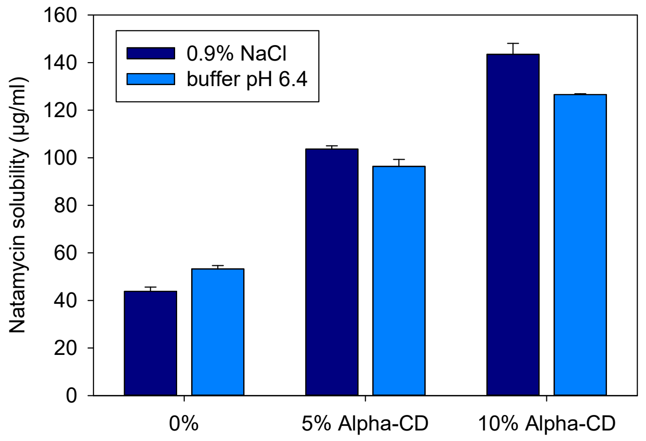

Figure S3. The apparent solubility of natamycin without αCD and containing 5–10% (*w*/*v*) in 0.9% NaCl and pH 6.4 buffer at 25 °C.

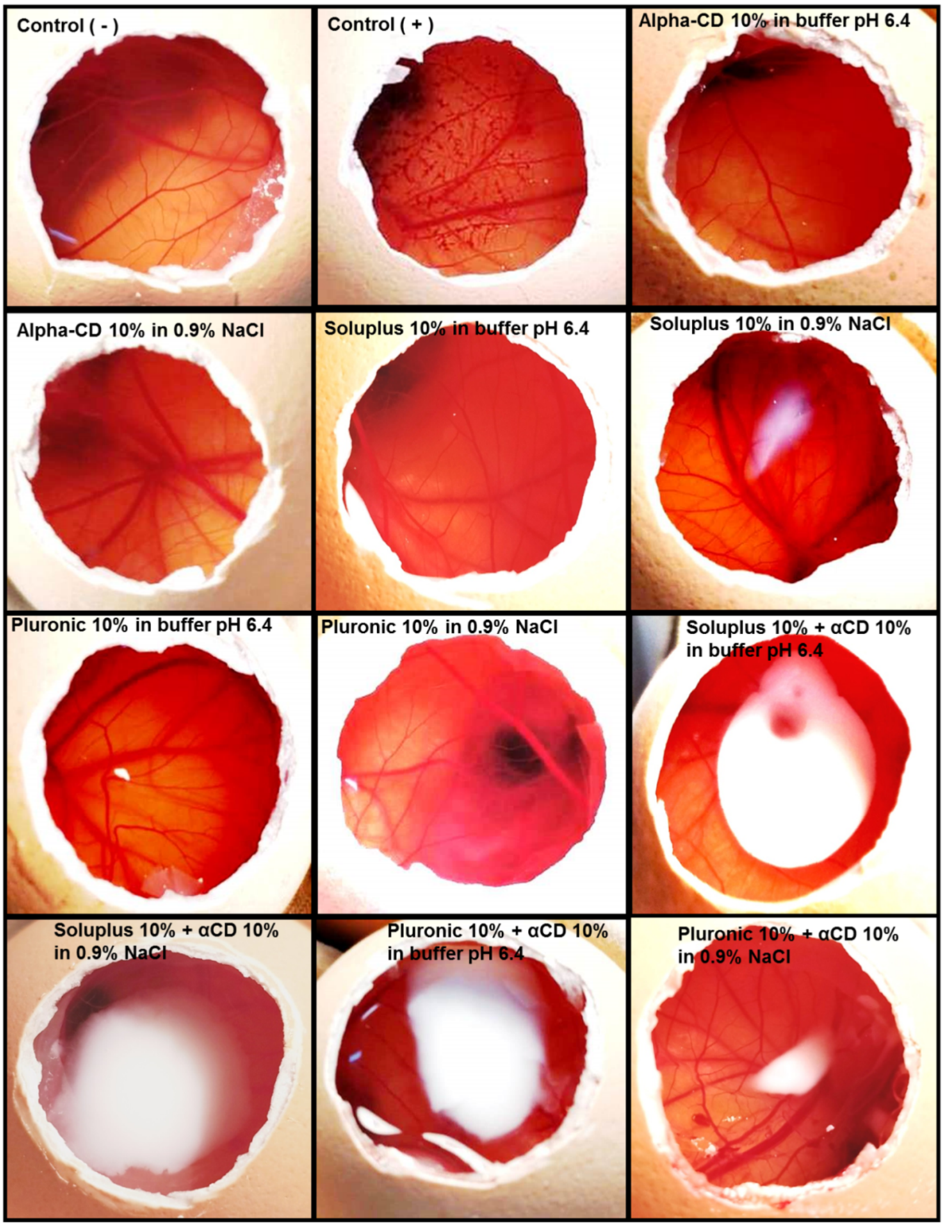

Figure S4. Pictures of Hen's Egg Test on the Chorioallantoic Membrane (HET-CAM) tests of Soluplus and Pluronic P103 formulations. Negative and positive controls refer to 0.9% NaCl and 0.1 N NaOH, respectively.
